# Intralesional meglumine antimoniate for the treatment of localised cutaneous leishmaniasis: a retrospective review of a Brazilian referral centre

**DOI:** 10.1590/0074-02760160183

**Published:** 2016-08

**Authors:** Rosiana Estéfane da Silva, Antonio Toledo, Maria Camilo Senna, Ana Rabello, Gláucia Cota

**Affiliations:** 1 Fundação Oswaldo Cruz, Centro de Pesquisas René Rachou, Centro de Referência em Leishmanioses, Pesquisas Clínicas e Políticas Públicas em Doenças Infecto-Parasitárias, Belo Horizonte, MG, Brasil; 2 Universidade José do Rosário Vellano, Faculdade de Medicina, Belo Horizonte, MG, Brasil

**Keywords:** cutaneous leishmaniasis, therapy, intralesional infiltration, antimoniate meglumine

## Abstract

Although intralesional meglumine antimoniate (MA) infiltration is considered an option for cutaneous leishmaniasis (CL) therapy and is widely used in the Old World, there have been few studies supporting this therapeutic approach in the Americas. This study aims to describe outcomes and adverse events associated with intralesional therapy for CL. This retrospective study reviewed the experience of a Brazilian leishmaniasis reference centre using intralesional MA to treat 31 patients over five years (2008 and 2013). The median age was 63 years (22-86) and the median duration time of the lesions up to treatment was 16 weeks. In 22 patients (71%), intralesional therapy was indicated due to the presence of contraindications or previous serious adverse events with systemic MA. Other indications were failure of systemic therapy or ease of administration. Intralesional treatment consisted of one-six infiltrations (median three) for a period of up to 12 weeks. The initial (three months) and definitive (six months) cure rates were 70.9% and 67.7%, respectively. Most patients reported mild discomfort during infiltration and no serious adverse events were observed. In conclusion, these results show that the intralesional MA efficacy rate was very similar to that of systemic MA treatment, and reinforce the need for further studies with adequate design to establish the efficacy and safety of this therapeutic approach.

Cutaneous leishmaniasis (CL) is a global health problem with no highly effective and minimally toxic therapy (González et al. 2009). In New World leishmaniasis, cutaneous lesions can be complicated by late mucosal involvement characterised by high morbidity and a lower cure rate, especially if caused by *Leishmania (V.) braziliensis*. In addition, a recently published literature review has confirmed that in the Americas, the spontaneous cure rate is low for CL (Cota et al. 2016). Due to these observations, the treatment of CL lesions is considered imperative. Pentavalent antimonial derivatives, such as meglumine antimony (MA), administered parenterally at a dose of 20 mg/kg/day for 20 consecutive days, is still the most studied and utilised treatment for CL; however, this approach can cause cardiac, hepatic, and renal toxicity. In 2010, the World Health Organization Expert Committee on Leishmaniasis recommended the inclusion of local and topical treatments among the acceptable therapeutic alternatives for New World leishmaniasis (WHO 2010). In 2013, the Pan American Health Organization Expert Committee on Leishmaniasis also included intralesional treatment in the regional guidelines restricted to reference centres and to single lesions not involving the face or joints (OPS 2013). Despite the efficacy being similar to that of systemic therapy with fewer adverse effects, evidence supporting this recommendation is limited. The aim of this study was to describe outcomes and adverse events associated with intralesional therapy for CL.

*Study design* - A retrospective study was performed based on the review of clinical records from patients who attended the Leishmaniasis Referral Centre of the Centro de Pesquisas René Rachou (CPqRR), a FIOCRUZ unit in Belo Horizonte, Minas Gerais, Brazil. In the present analysis, patients diagnosed with CL who submitted to MA intralesional treatment from January 2008 to December 2013 were included.

According to standard routine, CL diagnosis was established by direct smear, culture, or polymerase chain reaction (PCR). Besides the parasitological diagnosis, if other diagnostics were discarded, CL was also diagnosed based on presence of a suggestive lesion associated with a positive *Leishmania* intradermal skin test (Montenegro test). This retrospective study protocol was reviewed and approved by the CPqRR institutional ethical review board. During the study period, intra- lesional therapy was used as an alternative therapy for patients with localised disease and no mucosal involvement and for clinical or social conditions that did not allow the use of MA systemic therapy. At that time, there was no standardised protocol for the intralesional technique, except a biweekly infiltration schedule. The total infiltration volume corresponded to the amount required to achieve saturation of the lesion, at the time of full swelling. The infiltrations were interrupted when the lesion was completely healed or upon characterisation as therapeutic failure at a 90 day-follow-up. Meglumine antimoniate (Glucantime®, Aventis-Sanofi Pharma, São Paulo, Brazil) was supplied by the Brazilian Ministry of Health. Treatment outcomes were assessed using the set points and cure criteria proposed based on the current standardisation of outcomes in CL trials (Olliaro et al. 2013). Two different time points, from the first day of treatment, were evaluated for cure assessment, specifically day 90 ± 15 days for ‘‘initial cure” and day 180 ± four weeks for ‘‘definitive cure”. Cure was defined by complete re-epithelialisation of the ulcer, without any induration of the lesion site. As part of routine care in our service, haematological and biochemical tests, besides electrocardiogram, were performed for all patients receiving weekly treatments during follow-up. Cure assessment was performed by clinical examination. To assess adverse effects, all records and laboratory test results present in medical charts were evaluated. Descriptive statistical analysis of clinical variables was performed using SPSS software, version 10.0.

## RESULTS

From 2008-2013, 317 patients were diagnosed with CL in CPqRR. Thirty-nine patients (12.3%) received intralesional therapy during this period, and 16 (41%) were men and 23 (59%) women. CL diagnosis was confirmed by the identification of *Leishmania* through direct examination, culture, or PCR in 31 (79.5%) patients. In eight other patients (20.5%), diagnosis was defined by a positive *Leishmania* intradermal skin test plus the absence of other agents identified by parasitological tests plus a compatible inflammatory pattern upon histologic examination. Only parasitologically confirmed CL cases were included in this analysis and all relevant clinical data from these 31 patients are summarised in Table I *.* The median area of the ulcer lesions, which was the most frequent clinical presentation, was 1.7 cm^2^ (25-75% interquartile interval, 0.8-6.6 cm^2^). For most patients (58%), IL therapy was indicated due to the presence of one or more contraindications to systemic antimony treatment. In addition, in nine cases, MA intralesional therapy was used to treat lesions that were incompletely healed after systemic treatment (five patients) and four patients presenting serious adverse reactions to systemic antimony (four patients). Finally, in four other patients, the choice of intralesional therapy was at the request of the patient, and was motivated by schedule convenience or individual preference. Patients were treated with one-six MA intralesional infiltrations (84% patients received up to four infiltrations), during a period of up to 12 weeks with a volume of 1-10 mL of Glucantime® for each infiltration (Table II).

**TABLE I:** Characteristics of 31 patients with cutaneous leishmaniasis treated with intralesional meglumine antimoniate, Centro de Pesquisas René Rachou - Fundação Oswaldo Cruz (Fiocruz), Belo Horizonte, Minas Gerais, Brazil, 2016

Characteristic	Median (IR)
Age (years)	63 (41-76)
Lesion length before treatment (IR, weeks)	16 (8-28)
Lesion size (cm^2^)	1.7 (0.8-6.6)
Gender	n (%)
Male	15 (48)
Female	16 (52)
Disease type	n (%)
Primary cutaneous leishmaniasis	27 (87)
Relapsed cutaneous leishmaniasis	4 (13)
Number of lesions per patient	n (%)
one	22 (70.9)
two	6 (19.4)
three	3 (9.7)
Lesion location	n (%)
Head/neck	10 (32.3)
Arms/hand	10 (32.3)
Leg	7 (22.6)
Chest/back	4 (12.9)
Lesion type	n (%)
Ulcer	17 (54.8.0)
Papule	8 (25.8)
Plate	6 (19.4)
Intralesional therapy indication	n (%)
Systemic antimony contra-indication	18 (58.1)
Previous systemic antimony treatment failure	5 (16.1)
Serious adverse event with systemic antimony	4 (12.9)
Social or logistic reasons	4 (12.9)
Systemic antimony contra-indications*	n (%)
Elderly	13 (41.9)
Heart disease	10 (32.2)
Renal disease	6 (19.4)
Alcohol abuse	2 (6.5)
Enlarged QTc interval	2 (6.5)
Continuous use of medications that extend QTc interval	1 (3.2)
Liver disease	1 (3.2)

IR: interquartile range (25-75%); *: some patients presented more than one contraindication condition.

**TABLE II:** Intralesional infiltration therapy: details and outcomes of 31 patients with cutaneous leishmaniasis treated with intralesional meglumine antimoniate, Centro de Pesquisas René Rachou - Fundação Oswaldo Cruz (Fiocruz), Belo Horizonte, Minas Gerais, Brazil, 2016

Clinical or treatment data	Median (IR)
Volume of Glucantime infiltrated per session (IR, mL)	3.0 (1.8-4.4)
Total length of treatment* (IR, weeks)	4 (2-8)
Number of infiltration sessions	n (%)
one	6 (19.4)
two	11 (35.5)
three-four	9 (29)
five-six	5 (16.1)
Lost of follow-up	n (%)
three months	1 (3.2)
six months	4 (13.3)
12 months	8 (26.6)
Treatment response (intent-to-treat)	n (%)
Initial response (three month)	22/31 (70.9)
Definitive cure (six month)	21/31 (67.7)
Adverse events	n (%)
Eczema	2 (6.5)
Itching	5 (16.1)
Local edema	1 (3.2)
Intense pain	2 (6.5)
Malaise	2 (6.5)

IR: interquartile range (25-75%); *: treatment length of patients submitted to one infiltration was considered two weeks (time until the cure assessment).

At three months follow-up, 30 patients had their conditions evaluated; 22 had complete lesion healing and the absence of any inflammation, accounting for an initial cure rate of 70.9% (22/31) or 73.3% (22/30), excluding those lost from follow-up analysis. Six patients advanced with partial improvement (one patient presented an ulcer reduction above 50% of its initial area and five patients had complete ulcer re-epithelialisation, but retained inflammatory activity) and one patient presented with the emergence of a new skin lesion and received salvage therapy with amphotericin B. Six months after the beginning of intralesional therapy, 26 patients had their condition evaluated and 21 patients met the criteria of cure with complete ulcer epithelialisation and the absence of any inflammation in the lesion site. Therefore, according to the current recommended cure criteria and through the use of the intention to treat analysis (a more conservative approach), the definitive cure rate at six months was 67.7% (21/31) (Figure). If considering only the evaluated patients, the cure rate at six months was 77.7% (21/27). From the six patients presenting partial improvement at their three-month visit, two achieved lesion cure at their six-month visit. All four patients that were not cured at their six-month visit presented significant clinical improvement compared to their initial condition. One patient had an ulcer in its final healing phase, and the other three retained only mild local inflammation. These patients were observed with no further therapeutic intervention, and two were cured at their 12-month follow-up visit. The other two patients discontinued follow-up.

**Figure:**
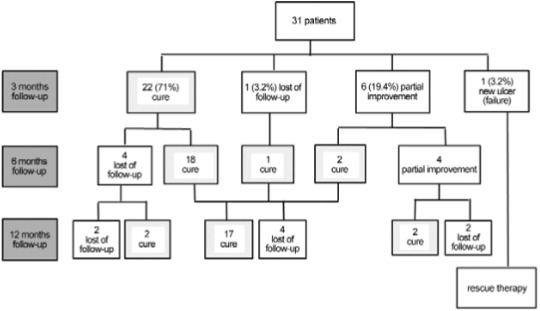
Follow-up and outcomes of 31 patients with cutaneous leishmaniasis treated with intralesional meglumine antimoniate, Centro de Pesquisas René Rachou - Fundação Oswaldo Cruz (Fiocruz), Belo Horizonte, Minas Gerais, Brazil, 2016.

Demographic and lesion characteristics (size and body localisation), previous antimony derivative therapy, therapeutic doses, and total length of treatment were not significantly different between cured and not cured patients. In contrast, previous systemic treatment failure was related to risk of failure with intralesional therapy (p = 0.05) based on univariate analysis. The number of follow-up patients lost at the 12-month visit was high (eight patients, 26.6%), hampering the efficacy analysis at this time point. However, it is important to highlight that no one-year relapse was observed among the patients cured at their six-month visit.

All patients reported mild discomfort during the infiltration session, and in two cases, it was described as intense pain. The adverse events identified were eczema (an intense inflammatory reaction), itching and swelling around the lesion, and malaise (Table II). No serious adverse events were described, and no patients presented mucosal lesions during the follow-up period.

## DISCUSSION

Intralesional therapy is a viable alternative for the treatment of cutaneous leishmaniasis in Brazil according to efficacy and toxicity data observed. There are a large percentage of CL patients that present contraindications to antimony systemic therapy (Vasconcelos et al. 2012), for whom other therapeutic approaches need to be developed. This group of patients consists mainly of older patients and those with comorbidities. In addition, several other circumstances, such as an incomplete clinical response or the occurrence of serious adverse events during systemic therapy, as well as social and health system conditions that impair the use of daily parenteral medication, justify the alternative use of topical therapies.

MA intralesional infiltration remains supported by weak evidence in the Americas. To best of our knowledge, there have only been two clinical prospective American studies addressing intralesional treatment, with only one being randomised (Oliveira-Neto et al. 1997, Soto et al. 2013). The main challenges include the lack of a standardised technique and the scarcity of local data regarding efficacy and safety. Available studies are based on different intralesional infiltration techniques with regard to infiltration volume, intervals, and total duration of treatment. Another unanswered question is the risk of late mucosal complications related to non-systemic treatments, which comprise intralesional infiltration, thermotherapy, cryotherapy and other topical therapies. Concerning this issue, it is important to remember that the late mucosal complication is described even after appropriate systemic treatment, which raises questions about the relevance of this concern (Blum et al. 2012). In addition, a further limitation is the requirement for a physician to perform infiltration in countries like Brazil, where invasive procedures are exclusive to medical professionals.

Potential advantages for intralesional infiltration include the use of lower total doses of antimony, a lower systemic level of tertiary antimony, and the possibility of using a more flexible schedule without the requirement of daily drug administration. It is important to highlight that systemic therapy with antimony requires clinical and laboratory monitoring and is difficult to perform in remote centres with little infrastructure. The cure rates herein described by using the intralesional approach are similar to those observed by others and resemble the rates obtained with systemic antimony derivative therapy (Tuon et al. 2008). These cure rates should be analysed with caution because they were obtained with an older population and with patients having other comorbidities that could negatively influence the therapeutic CL response, such as venous insufficiency and diabetes. In contrast, patients selected for intralesional therapy have few and small lesions, conditions that could favour healing. Furthermore, this review describes the results obtained using a non-standardised technique; therefore, some conditions, now discussed as essential for infiltration, such as saturation of the entire lesion, were not systematically utilised at that time.

Regarding adverse events, our experience confirms a low complication rate related to intralesional therapy. However, it is important to note the retrospective design of this study, which hampers a systematic analysis of adverse events. Eczema in the lesion site after MA infiltration occurred in two patients, and neither had been previously exposed to derivatives of antimony. This observation does not confirm the experience described by others (Ferreira-Vasconcellos et al. 2014), who hypothesised an association between this allergic reaction and prior sensitisation to antimony. Furthermore, one of the main limitations of this study was the lack of a standardised procedure for intralesional infiltration. This fact reflects a reality, even in reference centres, due to the lack of a validated intralesional infiltration technique. Based on its retrospective design and small number of patients, a thorough discussion about factors related to MA intralesional therapy failure could not be performed. However, the observed association between intralesional therapy failure and previous systemic therapy with MA requires more investigation and might suggest the presence of *Leishmania* spp. strains with reduced sensitivity to antimony. Our data should not be interpreted as evidence of efficacy, but rather as contributing to a novel hypothesis. Only through the use of a standard technique in different centres and a systematic surveillance protocol for possible adverse effects, will it be possible to compare results regarding the effectiveness and applicability of intralesional infiltration in our region. Based on these preliminary but encouraging results, our group is currently conducting a study to validate a standardised technique of intralesional infiltration and to perform a clinical trial addressing the efficacy and safety of a standardised MA infiltration technique for the treatment of localised CL. Unfortunately, the ideal trial design - a comparative study using systemic therapy with antimony as a control - would be difficult to implement since most eligible patients have contraindications to systemic therapy. At this point, our results confirm that prospective and well-designed studies should be conducted to assess CL intralesional therapy in the Americas.
